# NIR-Based Intelligent Sensing of Product Yield Stress for High-Value Bioresorbable Polymer Processing

**DOI:** 10.3390/s22082835

**Published:** 2022-04-07

**Authors:** Konrad Mulrennan, Nimra Munir, Leo Creedon, John Donovan, John G. Lyons, Marion McAfee

**Affiliations:** 1 Centre for Mathematical Modelling and Intelligent Systems for Health and Environment (MISHE), Atlantic Technological University, ATU Sligo, Ash Lane, F91 YW50 Sligo, Ireland; mulrennan.konrad@itsligo.ie (K.M.); nimra.munir@mail.itsligo.ie (N.M.); creedon.leo@itsligo.ie (L.C.); donovan.john@itsligo.ie (J.D.); 2 Centre for Precision Engineering, Materials and Manufacturing (PEM Centre), Atlantic Technological University, ATU Sligo, Ash Lane, F91 YW50 Sligo, Ireland; 3 Faculty of Engineering and Informatics, Technological University of the Shannon, Dublin Road, N37 HD68 Athlone, Ireland; sean.lyons@tus.ie

**Keywords:** PLA, NIR spectroscopy, soft sensor, bioresorbable polymer, PLS, random forest, support vector regression, chemometrics, extrusion

## Abstract

PLA (polylactide) is a bioresorbable polymer used in implantable medical and drug delivery devices. Like other bioresorbable polymers, PLA needs to be processed carefully to avoid degradation. In this work we combine in-process temperature, pressure, and NIR spectroscopy measurements with multivariate regression methods for prediction of the mechanical strength of an extruded PLA product. The potential to use such a method as an intelligent sensor for real-time quality analysis is evaluated based on regulatory guidelines for the medical device industry. It is shown that for the predictions to be robust to processing at different times and to slight changes in the processing conditions, the fusion of both NIR and conventional process sensor data is required. Partial least squares (PLS), which is the established ’soft sensing’ method in the industry, performs the best of the linear methods but demonstrates poor reliability over the full range of processing conditions. Conversely, both random forest (RF) and support vector regression (SVR) show excellent performance for all criteria when used with a prior principal component (PC) dimension reduction step. While linear methods currently dominate for soft sensing of mixture concentrations in highly conservative, regulated industries such as the medical device industry, this work indicates that nonlinear methods may outperform them in the prediction of mechanical properties from complex physicochemical sensor data. The nonlinear methods show the potential to meet industrial standards for robustness, despite the relatively small amount of training data typically available in high-value material processing.

## 1. Introduction

Polylactide (PLA) is a bioresorbable polymer derived from plant sources, which breaks down in vivo to H${}_{2}$O and CO${}_{2}$ over time. PLA undergoes biodegradation due to the presence of a hydrolysable backbone, which is subject to chemical hydrolysis in the presence of water at elevated temperature [1]. The biodegradation of PLA has made it an attractive material for packaging, textile, and medical applications. In medical applications, PLA has been used in temporary implantable devices such as bone fixation or local drug-delivery devices. PLA is one of the strongest FDA (Food and Drug Administration) approved bioresorbable polymers and so is of particular interest in applications where initial mechanical support is needed. The implant then degrades safely over time, possibly also releasing a drug such as an antibiotic or anti-inflammatory, as the host tissue heals. However, the industry faces issues regarding the processing of these polymers and their quality assurance once processed. The high temperatures and pressures required for forming the product also tend to degrade the material, which can lead to quality issues such as insufficient mechanical properties [2]. Identification and control of suitable manufacturing process conditions is extremely challenging, and can vary from batch to batch of raw material [3]. For applications requiring mechanical strength, the yield stress ($\sigma_{y}$) of the product is viewed as a critical quality characteristic. The laboratory characterisation of yield stress is a protracted, destructive test, and typically deficiencies in strength cannot be detected until several hours post-production, resulting in high scrap rates. Typical costs of medical or pharmaceutical grade PLA can be in the region of thousands of euros per kilogram, so this slow feedback on product quality results in an expensive process with limited production rates and risks in the reliability of supply.

Pressure and temperature sensors are routinely applied for continuous real-time monitoring of extrusion processes, but these provide limited information on physicochemical changes such as polymer degradation, which may occur during the manufacturing process. Over the last decade, many researchers have exploited the development of robust fibre-optic probes to apply vibrational spectroscopy techniques such as near-infrared (NIR), Raman, and UV–Vis, which are sensitive to changes in the molecular bonds present in polymeric materials while they are being extruded [4,5,6,7]. NIR is particularly attractive, as it is available at a lower cost than Raman but has greater sensitivity and specificity to molecular changes than UV–Vis. NIR spectra, however, have broad absorption bands and are dominated by overlapping overtone and combination band data and hence can be difficult to interpret. Usually, multivariate statistical approaches are applied to the spectral data in so-called ‘soft sensors’ to predict material properties which are measured offline. Due to the cost relative to conventional process instrumentation, NIR spectroscopy is most commonly applied in high-value polymer extrusion for monitoring of blends/mixtures. For example, its use has been widely reported for quantifying the drug content in pharmaceutical products under fixed processing conditions, using the classical chemometric regression technique of partial least squares (PLS) [8].

NIR spectral data contain a very high number of variables (absorbance of light at hundreds of wavelengths); however, the absorbances of adjacent wavelengths are highly correlated, and so a large number of variables are redundant. Hence, some form of feature reduction is required for regression. The classical approach is dimension reduction via a subset of features derived from a linear transformation of the original variables, such as in principal component regression (PCR) or PLS. PLS dominates as a soft-sensor method for spectroscopy data in polymer extrusion processes, with very little exploration of other methods in the field to date [8,9]. PLS has achieved a level of industrial acceptance due to its tried-and-tested effectiveness for chemometric applications, relative simplicity, and suitability for application with relatively small data sets, as is usually the case in process development for pharmaceutical and medical device products. However, PLS does not perform well where relationships in the data are nonlinear [10]. Further, PLS may in some cases reduce the access or the interpretability of the data, as information on which regions of the spectrum are responsible for the majority of the variation is obscured. Research studies in other fields have examined the use of PLS together with direct selection of a subset of the original wavelength variables (e.g., GA-PLS, bi-PLS, SiPLS), which can result in a simpler and more interpretable model [11]. However, these methods can be sensitive to the selection of training and validation data and may result in poor performance on new samples, especially where the training data set is small [12]. An alternative is to reduce the dimensionality of the data set by summarising intervals of the spectra with various statistics [12]; however, this adds significant complexity in processing the data and tuning additional hyperparameters such as the number of segments, which segments to retain in the regression model, and which statistics/features to use.

Nonlinear soft sensors using, e.g., artificial neural network (ANN) or kernel-based methods such as support vector regression (SVR) or Gaussian process regression (GPR) have commonly been applied in other fields and can outperform linear approaches [13,14]. With costly raw materials, such as in this application, the generation of training data is expensive, and hence methods which can be trained with relatively small amounts of data are required. Neural-network-based approaches typically require a large amount of training data; however, SVR is most commonly explored as a nonlinear alternative to PLS with spectral data and can perform well with a small number of samples [15,16]. Random forest (RF) regression has gained interest more recently for soft sensing with spectral data in industrial processes and appears to be promising for application with small amounts of data [17,18]. Zhang et al. [17] found that RF regression outperformed SVR and PLS in the quantification of multiple elements in 14 steel samples, using laser-induced breakdown spectroscopy (LIBS). Kneale and Brown [18] compared five soft sensor methodologies involving different implementations of PLS and RF regression on small data sets. The RF method, together with a hybrid RF–PLS method, (where PLS predictions are included as inputs to the RF regression model), offered the best performance on all data sets with non-monotonic response variables, and the hybrid model yielded the best one-step-ahead prediction on all data sets.

There has recently been increasing interest in monitoring/predicting parameters such as polymer degradation and mechanical properties in melt processing of bioresorbable and medical polymers. McKinley et al. [19] used ANN with an evolutionary algorithm to explore the effect of extrusion conditions on the mechanical properties of a drug-delivery vaginal film. However, this was to develop insight for process optimisation purposes rather than for quality monitoring. Montano-Herrera et al. [6] used NIR spectroscopy with PLS to predict the degree of thermal degradation induced during the extrusion of four different grades of the biodegradable polymer Polyhydroxyalkanoate (PHA). Muroga et al. [20] used NIR hyperspectral imaging on compression-moulded PLA samples to predict mechanical properties under different melting and annealing times and achieved reasonable predictions for the flexural properties and crystallinity of the samples using PLS regression. In this case, the NIR imaging was applied to room-temperature samples after they had been processed. In our previous work [21], we explored a low-cost method using only in-process pressure and temperature data for the prediction of yield stress in extruded PLA sheet using ensemble decision tree methods (bagging, random forest). If such soft-sensor approaches are to be used for quality assurance, then an appropriately rigorous performance evaluation is required to satisfy regulatory requirements. FDA guidelines for validation of qualitative analyses based on NIR models outline that the evaluation of such methods should include evaluation of the precision, accuracy, linearity, and robustness on an independent (‘external’) test data set [22]. None of the methods so far proposed have examined performance on an independent test set.

Hence, while both NIR and conventional pressure and temperature sensors appear to yield useful information on the subsequent mechanical properties of melt-processed PLA, the validity of using such sensor data for in-line monitoring of product quality in an industrial process has not yet been explored. It should be noted that in the previous NIR-based studies, process conditions were constant or the NIR imaging was performed on samples post-processing. However, NIR is sensitive to changes in process conditions including melt temperature, screw speed, and polymer rheology [23], and this may mask the effect of chemical changes in the material. In this paper we explore for the first time the application of both in-process NIR and pressure/temperature data to predict the mechanical properties of an extruded product and investigate whether such a sensing method can satisfy performance demands for quality assurance in the medical/pharmaceutical industries. As this involves spectral data, the modelling complexity significantly increases relative to our previous low-cost method (number of available input features increases from eight to several hundred). This is a significant challenge where it is too expensive and time-consuming to generate a large data set for model training (the so-called ‘curse of dimensionality’), and risks the development of a model with poor generalisation performance on independent data. Hence, in this work we compare the established regression algorithms for spectral data (PLS, PCR, ridge regression) to two promising nonlinear methods (SVR and random forest) and evaluate the performance based on FDA guidelines. This includes an independent external test set with perturbations in the processing conditions relative to the conditions used in the model training. A multi-rate data set comprising in-process NIR spectra together with data from pressure and temperature probes was captured from a small number of process trials covering a wide range of processing conditions under a design of experiments methodology. For the nonlinear methods, we explore a prior dimension reduction step via principal components (PC) analysis, where only the number of PCs to be included must be tuned. Dimension reduction via principal components is preferred here over methods which select a subset of the original variables, which, as discussed, are more interpretable but are too sensitive to the training/validation split in small data sets [12]. We show that it is possible to meet regulatory demands for accuracy and robustness, but only if NIR and pressure/temperature data are combined. Only the nonlinear methods preceded by a PC step satisfy the quality assurance requirements for use. The results significantly outperform those of earlier studies that used NIR imaging of post-processed PLA samples alone to estimate mechanical properties [20], with the advantage that the predictions are available continuously during processing, allowing corrective action to be taken if the product is not meeting specification.

## 2. Description of the Data Set

### 2.1. Experiments

**Figure 1:**
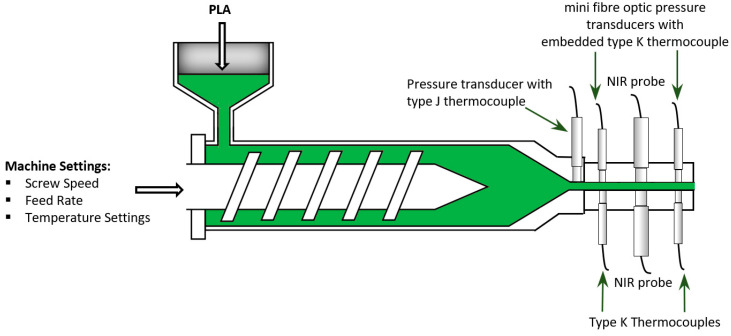
Schematic illustration of experimental set-up.

A Prism twin-screw extruder with four barrel zones was used along with a calender roll-off unit to manufacture extruded PLA sheet. The PLA grade was Ingeo^tm^ biopolymer 2003D from NatureWorks LLC. A slit die was attached to the end of the extruder and housed the sensors used to capture in-line process data. Data were recorded from pressure, temperature and NIR spectroscopic sensors. The pressure drop between two pressure transducers spaced along the slit die allowed for the shear viscosity of the material to be estimated during extrusion processing, as described in our earlier work [21]. Pressure and temperature data were acquired at sample rates between 5 and 10 Hz. A Dynisco pressure transducer with an embedded type J thermocouple was used along with two miniature, 3mm diaphragm, fibre-optic pressure transducers, each containing an embedded type K thermocouple. Two additional type K thermocouples that were flush mounted to the melt in the slit die were also used. NIR spectral data were captured in transmittance mode using a spectroscopy system provided by FOS Messtechnik GmbH that included an LR1 compact USB spectrometer with a resolution of 4 cm${}^{-1}$ (Aseq Instruments). Figure 1 shows a schematic overview of the experimental set-up. NIR spectra were measured in the 4000–7500 cm${}^{-1}$ wavenumber range with a spectrum typically collected every 30 s.

A design of experiments (DoE) methodology was applied to explore the effect of process conditions on the mechanical properties of the extruded PLA sheet within the processing window of the material. An initial set of 24 process trials consisted of twelve different processing conditions, each replicated using different combinations of extruder feed rate, screw speed, and temperature profile. The temperature profile included four barrel zones of the extruder (Z1, Z2, Z3, and Z4), the adaptor zone, and the die zone. The factor levels for the feed rate were 1160 g/hr (Low) and 1600 g/hr (High) and for screw speed were 56 rpm (Low) and 83 rpm (High). These were chosen at the extremities of the processing window for the material and extruder that was identified in the scoping trials. Three levels of temperature profile were applied: Low, Mid, and High, as presented in Table 1.

The initial process runs were full factorial, i.e., all combinations of factor levels were investigated with two replicates. The details of these experiments, together with their run order and factor level combinations are available in [21].

A second set of experiments comprising six process runs forming an independent test set, was conducted in a separate trial several months later. The factor levels for the feed rate and screw speed were the same as in the initial experiments; however, as presented in Table 2, slight changes were made to the temperature profiles to assess the robustness of the models to minor perturbations in process conditions. The process runs, the factor levels for each of the controllable variables, and their respective combinations are presented in Table 3.

Data were recorded for four minutes in all 30 runs once a steady state was reached (as indicated by a steady average pressure reading). Three samples were cut from the sheet extruded in each process run. The yield stress ($\sigma_{y}$) of each sample was measured using a Zwick Roell Z0.5 tensile tester with a load cell of 0.5 kN. Tests were carried out at a speed of 5 mm/min, with a measurement accuracy of ±1%. The mean yield stress for each process run was recorded.

### 2.2. Data Pre-Processing

As some regions of the NIR spectra were noisy and uninformative, only the region 6100–6700 cm${}^{-1}$, relating to C-H stretching overtones, was selected. This is the region most likely to be affected by the degradation of PLA. The spectral data were converted from transmittance to absorbance and pre-processed using multiplicative scatter correction and baseline correction. These pretreatments are well known and are described in detail in [24]. The sampling rate of the NIR system was lower than that of the pressure and temperature data, and the yield-stress response data were sampled at an even lower frequency (once per processing run). Typically, this situation is handled either by downsampling (to the lower frequency) or upsampling (to the higher frequency) [14]. Downsampling avoids introducing errors by interpolation of the low-frequency data, which may not result in a good representation of the true physical state. However, upsampling avoids any loss of information and can result in better soft sensor performance due to the larger data set available for modelling [25]. Here, as the process was monitored under steady-state conditions and the overall data set was small, we opted to upsample the data to preserve as much information as possible. A zero-order hold was applied for interpolation of the NIR data to the frequency of the pressure and temperature data (i.e., maintaining the most recent values at each time point until an updated spectrum arrived). The same value of mean yield stress was applied to all upsampled observations in each processing run. Following preprocessing of the data there were 50,762 observations (rows) and 612 features (columns) available for prediction of the product yield stress. The features included all pressure and temperature measurements, a shear viscosity estimate, and the NIR spectral data in the wavenumber range 6100–6700 cm${}^{-1}$. Each wavenumber in that range was a feature used for training the models, i.e., each wavenumber represented an input data column of observed amplitudes at that wavenumber.

## 3. Modelling Techniques

### 3.1. Linear Methods

Principal component analysis (PCA) is primarily used to reduce the dimensionality of data sets which contain a number of highly correlated features [26]. Highly correlated features may be largely redundant with respect to predictive ability, while high dimensionality tends to cause model overfitting. PCA performs a change of basis of the original data set to a new basis of orthogonal principal components (PCs). The PCs are uncorrelated linear functions of all original variables that successively maximise the explained variance in the data. Dimension reduction is achieved by choosing a subset of PCs that retains much of the variance of the original data set.

Principal component regression (PCR) uses a subset of these PCs in a linear regression model to predict the response *Y*. One of the problems associated with PCR is the selection of the optimum number of PCs. Generally, the first few PCs (major PCs), which capture most of the variability in the data, are selected, and the minor PCs are excluded in the final model. However, while the major PCs constitute the optimal low-order approximation of the input data set (minimum loss of information); there is no surety that these selected PCs are also relevant to the prediction of the dependent feature *Y* [27].

Partial least squares (PLS) regression also involves linear regression on a set of uncorrelated predictor features which are created from a linear transformation of the original set of variables. In PLS, the new features—the so-called ‘latent variables’—are formed from a simultaneous decomposition of *X* and *Y* such that these components successively maximise the covariance between the independent matrix *X* and the response feature *Y* [4]. As with PCR, a key problem is the choice of an appropriate number of features (latent variables or PCs). Selecting too few components may result in a regression model which does not explain all the relevant variance (underfitting), while selecting too many can lead to overfitting as noise is captured along with the systematic variance information.

Regularisation is a popular method used to solve the multicollinearity problem of linear regression, whereby the $X^{T}X$ matrix in the well-known least squares estimator Equation (1) is close to singular. In this case, slight variations in the data (such as adding or removing a few observations) will lead to significant changes in the coefficient estimates [28].

(1) $$\hat{\beta }={\left(X^{T}X\right)}^{-1}X^{T}Y$$

where $\hat{\beta }$ is the vector of estimated regression coefficients.

A regularisation method imposes a penalty on the size of the regression coefficients in the loss function of ordinary least squares (OLS). In ridge regression, a penalty proportional to the sum of the squares of the regression coefficients is added [29]. The loss functions for OLS and ridge regression are given in Equations (2) and (3) respectively, with *x* as an independent feature and *y* as a response feature.

(2) $$L_{ols}\left(\hat{\beta }\right)=\sum_{i=1}^{n}(y_{i}-x_{i}\hat{\beta }{)}^{2}$$

(3) $$L_{ridge}\left(\hat{\beta }\right)=\sum_{i=1}^{n}(y_{i}-x_{i}\hat{\beta }{)}^{2}+\lambda \sum_{j=1}^{m}\hat{\beta_{j}^{2}}$$

Here, *n* is the number of observations in the data set and *m* is the number of features in the model. λ is a penalty term which must be tuned.

As a result, a positive constant is added to the diagonal of $X^{T}X$ in the estimator equation, and the problem of matrix inversion of an ill-conditioned matrix is avoided. Ridge regression retains all the original features in the model but due to the penalty term λ, features which effectively constitute noise are shrunk towards zero, while the coefficients of highly correlated features are penalised.

An alternative regularisation technique is LASSO (least absolute shrinkage and selection operator), which imposes a penalty on the sum of the absolute magnitudes of the regression coefficients. The LASSO technique has some advantages over ridge regression in that it drives some of the coefficients to exactly zero, resulting in a more parsimonious model which may be more interpretable [30]. However, when features are highly correlated, LASSO tends to select just one of the features to have a non-zero coefficient, potentially resulting in some loss of information which may be useful for prediction. To overcome this limitation of LASSO, an ‘elastic net’ model combines the penalties on both the sum of absolute magnitudes and the sum of the squared magnitudes of the coefficients [30,31]. In this work, ridge regression outperformed both LASSO and elastic net in the accuracy of yield stress prediction, and therefore the other regularisation methods are not presented here.

### 3.2. Nonlinear Methods

In this study, we explored support vector regression and random forest regression as two methods which have been established to work well with small data sets in the handling of spectroscopy data. Support vector regression is an extension of the support vector machine algorithm [32]. The essential principle of SVR is the mapping of the data to a higher-dimensional space, where a linear regression is applied to give predictions within a defined margin of error from the true value. The dual slack variables ξ and $\xi^{*}$ are introduced in case there is no such function for which the constraints are feasible, and this allows for regression errors to exist beyond the margin (ϵ). This so-called ϵ-insensitive loss function means that any value with an error less than ϵ is ignored as zero, which tends to avoid overfitting of the model to the training data. The explicit mapping to the higher-dimensional space is avoided by means of the ‘kernel trick’, which reduces the computational load, i.e., the input (n×m) data matrix *X* is substituted by an (n×n) kernel matrix *K* which characterises an observation-to-observation relationship. Similar to ridge regression, the loss function for SVR (presented in the primal form in Equation (4)), also penalises the sum of squared magnitudes of the coefficient vector β, referred to as the model complexity. *C* is a positive constant which determines the trade-off between model complexity and the degree to which predictions outside the ϵ margin are tolerated. The optimisation problem is usually solved in the computationally simpler Lagrange dual formulation (see for example [33] for more in-depth reading on the SVR algorithm).

(4) $$L_{svr}\left(\beta \right)=\frac{1}{2}\beta^{\prime }\beta +C\sum_{n=1}^{N}\left(\xi_{i}+\xi_{i}^{*}\right)$$

The Gaussian radial basis function (RBF) given by Equation (5), is regarded as an ideal kernel choice to model functions of arbitrary nonlinearity [34]. The resulting SVR model hyperparameters therefore include the error tolerance margin (ϵ), the trade-off parameter *C*, and the Gaussian kernel parameter γ.

(5) $$K\left(x_{i},x_{j}\right)=\exp\left(-\gamma {∥x_{i}-x_{j}∥}^{2}\right)$$

Random forest (RF) is an ensemble learning method whereby several decision trees are grown using bootstrapped data [35]. The regression predictions are the averages of all the decision tree predictions. RF differs from a bagging (bootstrap aggregation) model in using a tuning parameter *m${}_{try}$*. The parameter *m${}_{try}$* is a number controlling the size of the subset input features sampled at random at each node of the RF decision tree for its split decision. The model then searches for the best feature and split value from within that subset. By selecting an *m${}_{try}$* value which is less than the total number of input features, only a subset of input features is considered at each split. This is known as decorrelation of the trees and results in a variance reduction in the model. This is achieved because averaging a number of uncorrelated features results in greater variance reduction than averaging a number of positively correlated features. The algorithm is described in the following steps:

1. Choose the total number of trees to grow in the random forest (*T*).
2. Choose a value for $m_{try}<$ the number of input features.
3. For t=1 to *T*:
  (a) Generate a bootstrap data set from the training data.
  (b) Grow a random forest regression tree $R_{t}$ for the bootstrapped data, by recursively repeating the following steps for each terminal node of the tree, until the stopping criterion is reached.
    (i) Select $m_{try}$ input features at random as candidates for splitting the node.
    (ii) Pick the best feature/split-point among the $m_{try}$, which minimises the combined residual sum of squares of the two subsequent nodes.
    (iii) Repeat until reaching terminal nodes that have ≥5 observations. If a node has >5 observations, it becomes a terminal node if a split will leave subsequent nodes with <5 observations.
  (c) Repeat until *T* trees are grown.
4. Output the ensemble of trees {$R_{1},R_{2},\ldots ,R_{T}$}.
5. Average the predictions from each regression decision tree to predict using the forest.

## 4. Model Training

The data split for training and testing the models was prepared as follows. The data from the 24 initial experiments were split in a ratio of 65:35. The 65% set was used as the training data for each of the models. The 35% set, referred to as the ‘internal validation’ set (referencing the language in the FDA guidance for NIR-based soft sensors [22]), was used to tune the model hyperparameters. The data from the six processing runs in the second set of experiments were withheld as an independent ‘external’ test set. Model training involved the minimisation of the root mean squared error (RMSE) over all upsampled observations (as described in Section 2) in the training set. The model hyperparameters were then tuned according to the lowest RMSE on the internal validation set.

For PLS and PCR, the number of components included was increased in increments of 1 until a clear minimum in the RMSE on the internal validation set was identified. PLS performed optimally on the internal validation set with 30 components, while the optimal PCR model had 290 components (from the original set of 612 features). For ridge regression, λ was varied between 0 and 3 in increments of 0.01. The lowest RMSE on the internal validation set was achieved with λ=0.41.

Multiple SVR models were trained using original features or principal components. An SVR model using the original features was trained using default parameters, i.e., ϵ=0.1, C=1, and $\gamma =\frac{1}{n_{f}}$, but did not generalise well to the external test set, despite having reasonable performance on the internal validation data. The term $n_{f}$ represents the number of training features. Further hyperparameter tuning was not pursued using the original features. The principal components were then used to train several SVR models. A bottom-up approach was taken to determine the optimal number of PCs, i.e., beginning with the first five PCs, a model was trained, and on each iteration the subsequent five PCs were added. It was discovered that an SVR model using the first 25 PCs was best. The hyperparameters ϵ and *C* were tuned, while γ remained at the default value, which was equal to 0.04 for 25 PCs. The optimised PCA–SVR model had ϵ=0.05 and C=0.25.

Random forest models were trained using all 612 original features, all PCs, or subsets of PCs. For each of the models, either with original features or with all PCs, an *m${}_{try}$* value equal to 204 was used, i.e., one third of the number of features, which is the default value. To reduce the dimensionality, the model performance with a subset of PCs was investigated, similarly to the approach used in the SVR model training. The number of PCs to include and the model *m${}_{try}$* value were both tuned in a two-step process. First, an RF model was trained for each of a different number *n${}_{PCs}$* of the major PCs. Values of *n${}_{PCs}$* of 5, 10, 15, 20, 30, and 50 were tested. One hundred trees were used in each model, where an *m${}_{try}$* value equal to, or rounded up from, $\frac{n_{PCs}}{3}$ was applied. This is an accepted heuristic [35]. The models with 15, 20, and 30 PCs had similarly low RMSE values on the internal validation set; however, the model with 20 PCs performed best on the external test set. Hence, PCs 1 to 20 were chosen as the subset of features to fit the final model. In the second step, using the subset of 20 major PCs, all possible models with 100 trees were trained by varying the tuning parameter *m${}_{try}$* from 1 to 20. The number of trees was fixed at 100 as it was observed that there was no performance gain above that threshold. An *m${}_{try}$* value of 5 resulted in the lowest RMSE value on the internal validation set, and this model was selected for performance evaluation.

It is worth noting that we also investigated whether there was an advantage to using an RF model to select a subset from all the PCs (not just the major ones), as an alternative feature selection step. Here, an RF model was trained using all 612 PCs, and an internal feature importance score was generated by the model. The model ranked features by permuting the last feature in each subset used to split tree nodes and measuring the effect on the model error during the training phase. Those which had the greatest effect on the model error were ranked in order of importance. RF models were then generated using each of the top 30 and top 50 important features from the model ranking, and the heuristic *m${}_{try}$* value of $\frac{n_{PCs}}{3}$ was applied. Neither of these models outperformed the the PCA–RF soft sensor using only the first 20 major PCs, and therefore we felt there was no advantage to this approach, which was significantly more time-consuming than selecting from the major PCs.

## 5. Performance Evaluation

FDA guidelines endorse the use of predictive models to realise the potential of in-line analytical sensors for quality assurance, provided the model reliability is adequate for the purpose [22]. Assessment of the reliability should include the consideration of: accuracy (predictive errors of calibration and independent test data as well as biases in prediction); precision (standard deviation of predictions); linearity (of predicted vs. measured values over the full range of the analytical procedure); and robustness (e.g., to variations in environmental conditions, operating conditions, raw materials, etc.). Here, we treat the initial 24 experimental process runs, which cover the full process window of the material, as the calibration data set and assess the calibration performance on the internal validation data. Experimental runs 25–30 were conducted months later, allowing for variations in the environmental conditions and raw material as well as introducing variations in the temperature settings relative to the calibration runs. These six later runs were used as an external test set and allowed for an assessment of robustness. Note that similar studies on the validation of soft sensors for monitoring drug content using in-process NIR, Raman, or UV–Vis spectra typically have internal validation sets of fewer than ten runs and external test sets of the order of three to five runs (e.g., [5,36]).

Model accuracy was evaluated based on the mean predicted value of the yield stress for each process run, as given by Equation (6). That is, while a yield stress prediction was made for every observation in the process data (at a frequency of 5–10 Hz), the observed mean yield stress in the product was indicative of the material that had been processed over the entire four-minute run at each process condition. Hence, by averaging the predictions over the equivalent period, a more appropriate rate for predicting mechanical properties was applied. In the equation below, $\hat{y_{i}}$ is the mean predicted value of the yield stress and $\bar{y_{i}}$ is the mean value of the yield stress observed for the corresponding process run, while ($n_{r}$) is the number of process runs.

(6) $$RMSE=\sqrt{\frac{\sum {\left(\hat{y_{i}}-\bar{y_{i}}\right)}^{2}}{n_{r}}}$$

**Figure 2:**
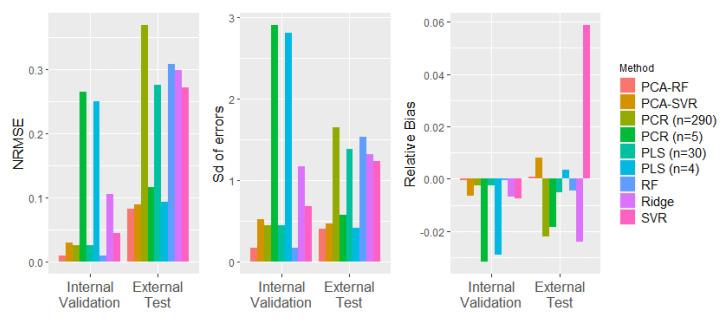
NRMSE, SD of errors, and relative bias for all soft sensors.

**Figure 3:**
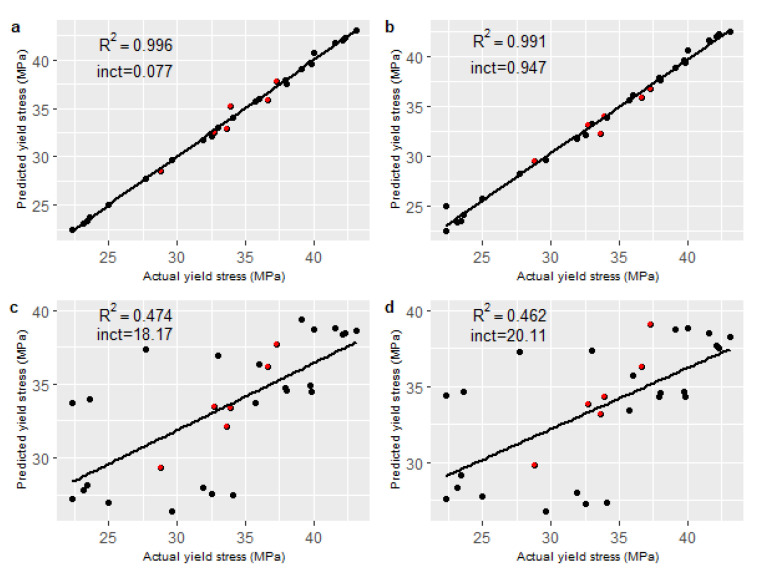
Linearity plots with R^2^ and intercept values for all unseen data. (**a**) PCA–RF; (**b**) PCA–SVR; (**c**) PLS (n = 4); (**d**) PCR (n = 5). The black points relate to the internal validation set and the red points relate to the external test set.

Table 4 lists the *RMSE* values of all models for the internal validation set and the external test set. A normalised root mean squared error (NRMSE) was also calculated by dividing the *RMSE* by the range of the mean yield stress values in the data set to illustrate the magnitude of the errors relative to the range of variation in the true values. The NRMSE is illustrated for all models in Figure 2, together with the standard deviation (Sd) of the model errors and the relative bias of the predictions. Note that while high numbers of components gave the best performance for both PCR and PLS on the internal validation set, these models performed poorly on the external test set and were clearly overfitting to the data captured in the initial 24 experiments. In similar applications, performance on the external test set is used to select the number of components (e.g., [36]). Here, best performance on the external test set was achieved with a PCR model with five PCs and a PLS model with four latent variables. This resulted in lower external test errors than internal validation errors, as is also seen in other studies using this approach [36]. In this case, the number of model components selected was tuned to the ’external’ test set such that it was no longer truly independent. High errors on several of the process runs in the internal validation data were evident in the linearity plots for PCR and PLS (Figure 3c,d), giving poor confidence in the reliability of these models over the full range of processing conditions. The PCA–RF hybrid model outperformed all other models on both sets of unseen data for all error metrics.

Model linearity was determined by plotting the predicted yield stress against the measured yield stress, and a coefficient of determination ($R^{2}$) value and y-intercept was calculated for each model, as shown in Figure 3. It can be seen that both the PCA–RF and the PCA–SVR model exhibited excellent linearity relative to the conventional linear methods.

**Figure 4:**
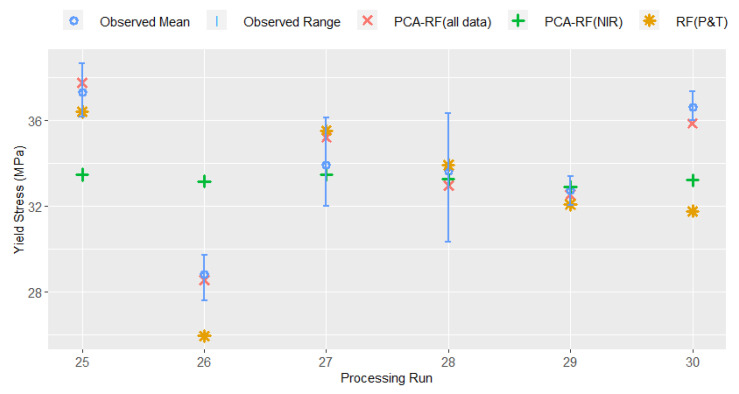
Comparison of predictions for external test set using: NIR and pressure and temperature data; pressure and temperature data only; and NIR data only.

As outlined in the Introduction, our previous work examined the use of low-cost pressure and temperature instrumentation for the prediction of yield stress in PLA extrusion [21]; however, this method was not previously analysed for robustness on an external test set. The RMSE of the PCA–RF model proposed in [21] on the internal validation and external test sets is also reported in Table 4, and it is evident this demonstrates poor accuracy on the external test set. The performance of an RF model (without a prior PC step), as well as PCR and PLS models on the same data set are also given for comparison. The ability to predict product yield stress using NIR data alone was also investigated for the RF, PCA–RF, PCR and PLS models (with hyperparameters tuned in the same fashion as for the full data set described above). All models based solely on NIR data performed poorly on both the internal validation and external test sets. As NIR is known to be sensitive to process conditions, particularly temperature, it is unsurprising that using NIR data without information on the physical state of the melt is insufficient to predict the mechanical properties of the extruded product. The predictions of the best-performing model (in all cases a random forest model) on each of the subsets of sensor data are shown with the range and mean of the observed yield stress values for the external test set in Figure 4.

## 6. Discussion and Conclusions

The use of in-process vibrational spectroscopy tools coupled with intelligent sensing concepts is gaining acceptance for product quality monitoring in extrusion of medical and pharmaceutical products; however, validated applications are so far limited to quantifying mixture concentrations (e.g., drug content), with fixed processing conditions and almost always using the linear method of partial least squares. In this paper, we showed that combining in-process NIR with pressure and temperature sensors can allow reliable prediction of the mechanical properties of a bioresorbable polymer product under different processing conditions. While previous work indicated the potential of using low-cost pressure and temperature instrumentation for soft sensing of mechanical properties in PLA extrusion, it was shown here that model robustness is significantly improved with the addition of NIR data, which provides information on molecular bond activity in the polymer melt as well as information on the physical state (temperature, viscosity, etc.). The results of the low-cost method may be satisfactory for process control in non-critical applications such as biodegradable packaging, but they are not sufficient to satisfy quality assurance demands in highly regulated industries. Due to the cost of the NIR equipment, the method proposed here is most suitable for high-value PLA applications with stringent quality demands, such as in pharmaceutical and medical device products. Further, the approach described here performs significantly better on predictive accuracy relative to other studies that predicted the mechanical properties of PLA using PLS regression applied to hyperspectral NIR images of post-processed samples [20]. Imaging of post-processing samples at room temperature removes the complication of the sensitivity of the NIR spectra to changes in processing temperature, melt viscosity, etc. However, a further advantage of the approach presented here is that the mechanical property predictions are available in real time during processing, and are available continuously rather than only on post-processing samples of the product.

We showed that a nonlinear regression method is needed in this application in order to satisfy the reliability demands outlined in regulatory guidelines for quality analysis in the medical and pharmaceutical industries. Both support vector regression and random forest regression approaches worked well when preceded by a principal component dimension reduction step. While the PCA–RF approach gave the best performance, further tuning of the PCA–SVR method is possible. However, an advantage of the random forest method is the simplicity in model tuning, where only the number of PCs to be included and a single hyperparameter (*m${}_{try}$*) require tuning, compared to SVR where the number of PCs and three hyperparameters require tuning. However, it should be noted that other nonlinear methods have also been proposed in the literature and may also perform well for such a task. In particular, kernel-PLS has been found to have a similar performance to SVR and may be a more attractive alternative in the industry, which has a level of familiarity with PLS methods and their interpretability [10].

A significant constraint on model development in medical and pharmaceutical applications is the cost of generating training data using very high-value raw materials; however, the nonlinear methods proposed here perform well against all industry-standard reliability metrics for analytical procedures, even with the constraint of limited training data. Further reliability testing should be carried out if the method is to be applied outside the range of conditions analysed in the external test set used here. However, the results indicate the potential for in-process measurements to give rapid feedback for process control purposes, and may reduce the amount of off-line destructive testing of the product required for quality assurance purposes. A challenge to industrial acceptance in highly conservative, regulated industries such as medical devices is the ’black box’ nature of such models. The interpretability of nonlinear approaches should be addressed in future work.

## Figures and Tables

**Table 1:** Factor levels for the temperature profile in runs 1–24 (used in training).

Factor Level	Temperature Profile (°C)					
	Z1	Z2	Z3	Z4	Adaptor	Die
Low	130	190	200	200	200	200
Mid	130	190	200	205	210	210
High	130	190	200	210	220	220

**Table 2:** Factor levels for the temperature profile in runs 25–30 (independent external test set).

Factor Level	Temperature Profile (°C)					
	Z1	Z2	Z3	Z4	Adaptor	Die
Low	130	180	200	200	200	200
Low-Mid	130	180	200	200	205	205
Mid	130	180	200	200	205	210
High	130	180	200	200	210	220

**Table 3:** Factor levels (SS = screw speed; FR = feed rate; TP = temperature profile) for runs 25–30 (independent external test set).

Process Run	SS Level	FR Level	TP Level
25	Low	High	Low
26	High	Low	High
27	Low	High	Low-Mid
28	Low	High	Mid
29	Low	Low	Low-Mid
30	High	Low	Low

**Table 4:** Soft-sensor RMSE values.

RMSE		
Soft Sensor	Internal Validation	External Test
**All sensor data**		
PCA–RF	0.2071	0.704
PCA–SVR	0.627	0.757
PLS (n = 4)	5.207	0.796
PCR (n = 5)	5.525	0.994
SVR	0.923	2.315
PLS (n = 30)	0.545	2.346
Ridge λ = 0.41	2.191	2.543
RF	0.1965	2.622
PCR (n = 290)	0.541	3.141
**Pressure and temperature data only**		
RF	0.597	2.439
PLS (n = 2)	5.38	2.631
PCR (n = 3)	5.369	2.714
PCA–RF [21]	0.185	5.026
**NIR data only**		
PCA–RF	6.839	2.758
PCR (n = 2)	7.06	2.789
PLS (n = 2)	6.980	2.813
RF	5.875	3.121

## Data Availability

The data presented in this study are available on request from the corresponding author.
